# Constructing hypoxia and bioshield inhibits neurovascularized endochondral ossification for stable cartilage regeneration

**DOI:** 10.1016/j.bioactmat.2026.08.030

**Published:** 2026-10-10

**Authors:** Bao-xing Tian, Hui-lin Sun, Bao-shuai Bai, Chen Jin, Qian-yi Wang, Guang-dong Zhou, Xiao-xi Lin, Bin Wang, Dong Lei, Meng-jie Hou

**Affiliations:** a Department of Laser and Aesthetic Medicine, Shanghai Ninth People's Hospital, Shanghai Jiao Tong University School of Medicine, Shanghai, 200011, PR China; b Department of Plastic and Reconstructive Surgery, Shanghai Key Lab of Tissue Engineering, Shanghai Ninth People's Hospital, Shanghai Jiao Tong University School of Medicine, Shanghai, 200011, PR China; c Department of Plastic Surgery, The Second Affiliated Hospital of Zhejiang University College of Medicine, Hangzhou, 310000, PR China

**Keywords:** Cartilage regeneration, BMSCs, Ectopic microenvironment, Neurovascularization, Endochondral ossification

## Abstract

Cartilage regeneration by bone marrow stromal cells (BMSCs) has become a promising therapeutic approach. However, the BMSC-engineered cartilage (BEC) is highly prone to ossification once implanted in vascularized and innervated subcutaneous microenvironments, ultimately leading to failure of cartilage regeneration. Herein, we innovatively developed a living cell-based encapsulation coating technique (designated as EC@GelMA) aimed at creating a biophysical barrier for BMSCs to maintain their stability in cartilage regeneration within an ectopic non-neurovascularized microenvironment. *In vitro* experimental results confirm that, both technically and theoretically, EC@GelMA could provide a hypoxic and biologically active biomimetic cartilage microenvironment for internally encapsulated BMSCs. *In vivo* experiments confirmed that EC@GelMA can provide BMSCs with a biomimetic cartilage microenvironment that isolates blood vessels and nerves, achieving stable cartilage regeneration of BMSCs in the ectopic microenvironment. All our results provide an effective solution for repairing cartilage defects in subcutaneous ectopic microenvironments, such as ear and nasal cartilage, using BMSCs, and facilitates its clinical translation.

## Highlights

• **Development of a living cell-based encapsulation coating (EC@GelMA)** that creates a biomimetic, multi-layered protection system featuring both hypoxic and biological barriers for BMSC-based cartilage regeneration.
• **Elucidation of the dual-mechanism:** The system synergistically combines initial physical shielding with long-term biological regulation, actively maintaining a pro-chondrogenic and anti-ossification microenvironment.
• **Demonstration that this technology effectively isolates host blood vessels and nerves in vivo**, physically blocking the key extrinsic factors that drive endochondral ossification in ectopic sites.
• The proposed “material-cell” synergy paradigm is a **generalizable strategy** that can potentially be adapted with other cell types or hydrogels to address tissue regeneration challenges where maintaining a stable cell phenotype against adverse environmental cues is critical.

## Graphical abstract

**Figure:**
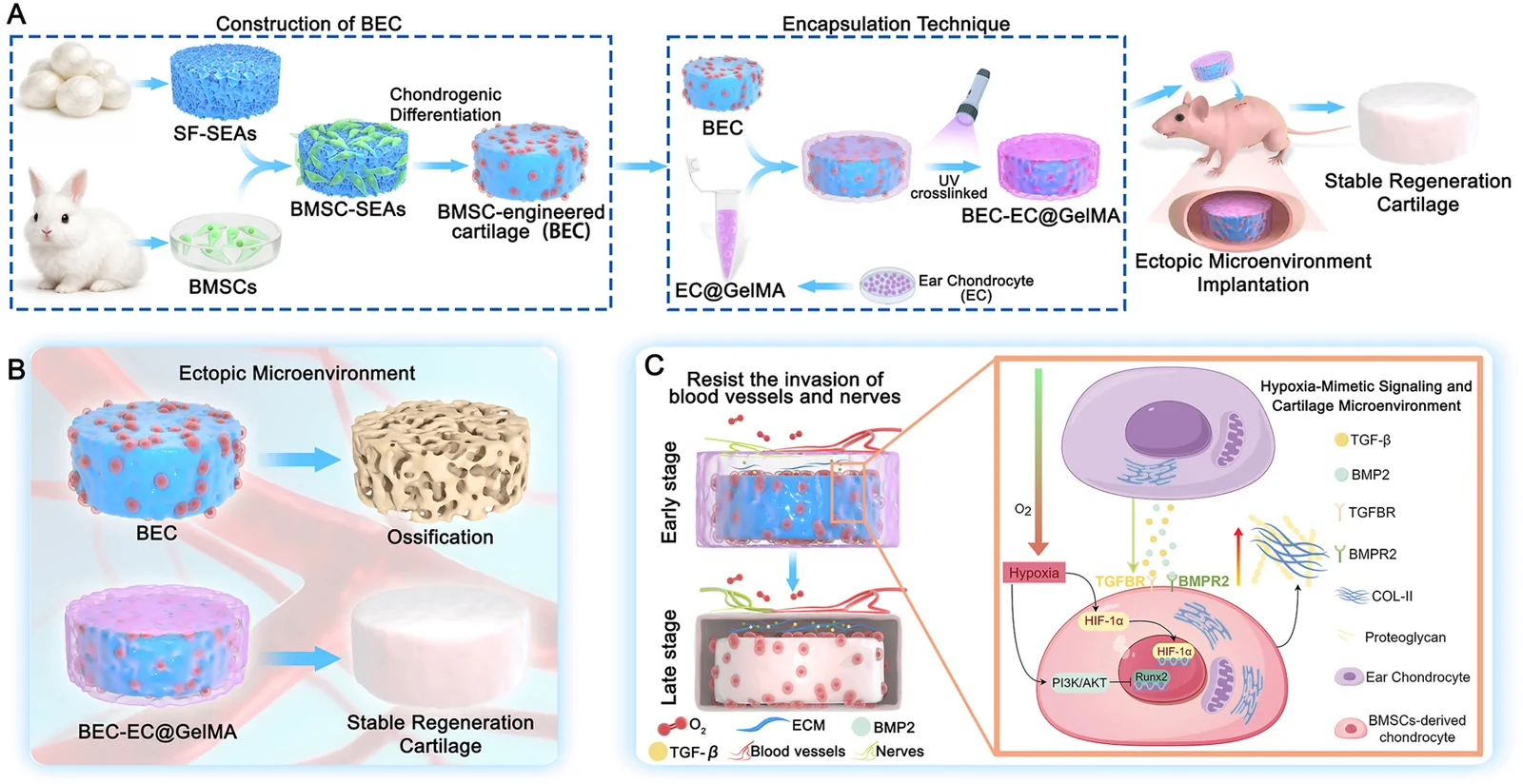

## 1 Introduction

Cartilage is highly susceptible to irreversible damage caused by various diseases and injuries, often leading to significant functional impairment. Owing to its avascular and aneural nature, cartilage exhibits an intrinsically limited capacity for self-repair. Tissue engineering is a promising approach for cartilage regeneration, in which seed cells serve as the core element and a major factor determining the clinical translation of tissue-engineered cartilage [1]. BMSCs are considered as ideal seed cells due to their wide range of sources, self-renewal, and multi-directional differentiation [[2], [3], [4]], and have been widely used in the regeneration of cartilage *in vitro* and the repair of articular cartilage defects *in vivo* [[5], [6], [7]].

However, BMSC-engineered cartilage (BEC) is highly prone to ossification once implanted in vascularized and innervated subcutaneous microenvironments (such as those involved in the repair of auricular or nasal cartilage defects), ultimately leading to failure of cartilage regeneration [8,9]. The specific process is that within the hostile *in vivo* microenvironment, the engineered cartilage often undergoes aberrant remodeling, progressively replaced by bone tissue. This process is largely initiated by host-derived vascular invasion, which not only facilitates nutrient exchange but also delivers osteoinductive signals [[10], [11], [12]]. Subsequent infiltration by blood vessels and nerves further disrupts the cartilaginous niche, promoting terminal chondrocyte differentiation into hypertrophic cells, culminating in calcification and ossification [[13], [14], [15]]. This aberrant phenotypic transition represents a fundamental biological barrier that must be overcome to enable the successful clinical application of engineered cartilage therapies. This remains the main reason why BMSCs have not yet achieved widespread clinical translation for cartilage regeneration applications.

In recent years, several strategies have been developed to address the challenge of ossification during cartilage regeneration by BMSCs in ectopic microenvironments. The main purpose is to construct a microenvironment that isolates blood vessels and nerves for BMSCs implanted in the ectopic microenvironment, thereby promoting stable cartilage regeneration. Many researchers initially adopted mixing BMSCs with chondrocytes and then implanting it into the ectopic microenvironment *in vivo* [[16], [17], [18]]. However, it faces several key limitations. Firstly, the random distribution of cells in co-culture leads to inconsistent paracrine signaling and uneven cell-to-cell contact, resulting in heterogeneous differentiation outcomes. In such conditions, some BMSCs escape chondrogenic induction and remain susceptible to osteogenic cues from the host microenvironment. Secondly, its efficacy is highly dependent on a high proportion of chondrocytes, typically requiring 30%-50% or even greater ratios to achieve reliable inhibition. Given the limited availability of primary chondrocytes *in vitro*, this poses a fundamental translational barrier. Furthermore, recent advances have employed electrospun membranes loaded with anti-angiogenic agents (such as axitinib or bevacizumab) to encase BEC, aiming to create a pharmacological shield against vascular invasion and subsequent ossification [19,20]. Although the methods can to some extent inhibit the ossification of BMSCs in ectopic environments, it still faces key limitations: electrospun membranes loaded with anti-angiogenic drugs face key limitations in preventing ectopic ossification: (1) Their action is passive, only blocking blood vessels without actively guiding BMSC differentiation; (2) drugs may have side effects and release unevenly over time; (3) protection ends once the drug runs out, risking long-term failure. Therefore, there remains a compelling need for a strategy that can not only provides a durable physical barrier against host-derived inductive signals, but also actively and continuously guides BMSCs toward a stable chondrogenic phenotype, while overcoming the limitations of cell sourcing and pharmacological dependency.

To address the aforementioned challenges, based on our group previous studies [7,21,22], in the current study, we innovatively developed a living cell-based encapsulation coating technology, designated EC@GelMA (Fig. 1). This technology involves encapsulating chondrocyte coatings on the surface of BEC constructed *in vitro* before it is implanted into the ectopic microenvironment *in vivo*, thereby verifying whether this technology can maintain the stability of cartilage regeneration of BMSCs in the ectopic microenvironment. Firstly, silk fibroin (SF) suitable for cartilage regeneration was prepared using techniques established in the previous studies and BEC was constructed. The simple hydrogel and the mixture of ear chondrocytes/hydrogel were respectively coated on the surface of the BEC according to the explored appropriate number of layers. The results of transcriptome sequencing indicated that compared with the uncoated group, the HIF-1 signaling pathway was significantly activated in both the chondrocyte-coated encapsulation group. Furthermore, *in vivo* experiments confirmed that the chondrocyte coating encapsulation technology can provide BMSCs with a biomimetic cartilage microenvironment that isolates blood vessels and nerves, achieving stable cartilage regeneration of BMSCs in the ectopic microenvironment. These findings show that the EC@GelMA encapsulation system creates a biomimetic, multi-layered protective barrier with both hypoxic and biological features, blending instant physical shielding with sustained biological regulation. The GelMA hydrogel of EC@GelMA provides an initial physical barrier against host invasion, while the encapsulated ECs act as sustained bioactive units. Even after GelMA degradation, these units can actively establish and maintain a pro-chondrogenic, anti-angiogenic, and anti-neurogenic chondroprotective microenvironment via paracrine signaling, thereby enabling stable cartilage regeneration in ectopic microenvironments. This provides an effective solution for repairing cartilage defects in subcutaneous ectopic microenvironments, such as ear and nasal cartilage, using BMSCs, and facilitates its clinical translation.

**Fig. 1:**
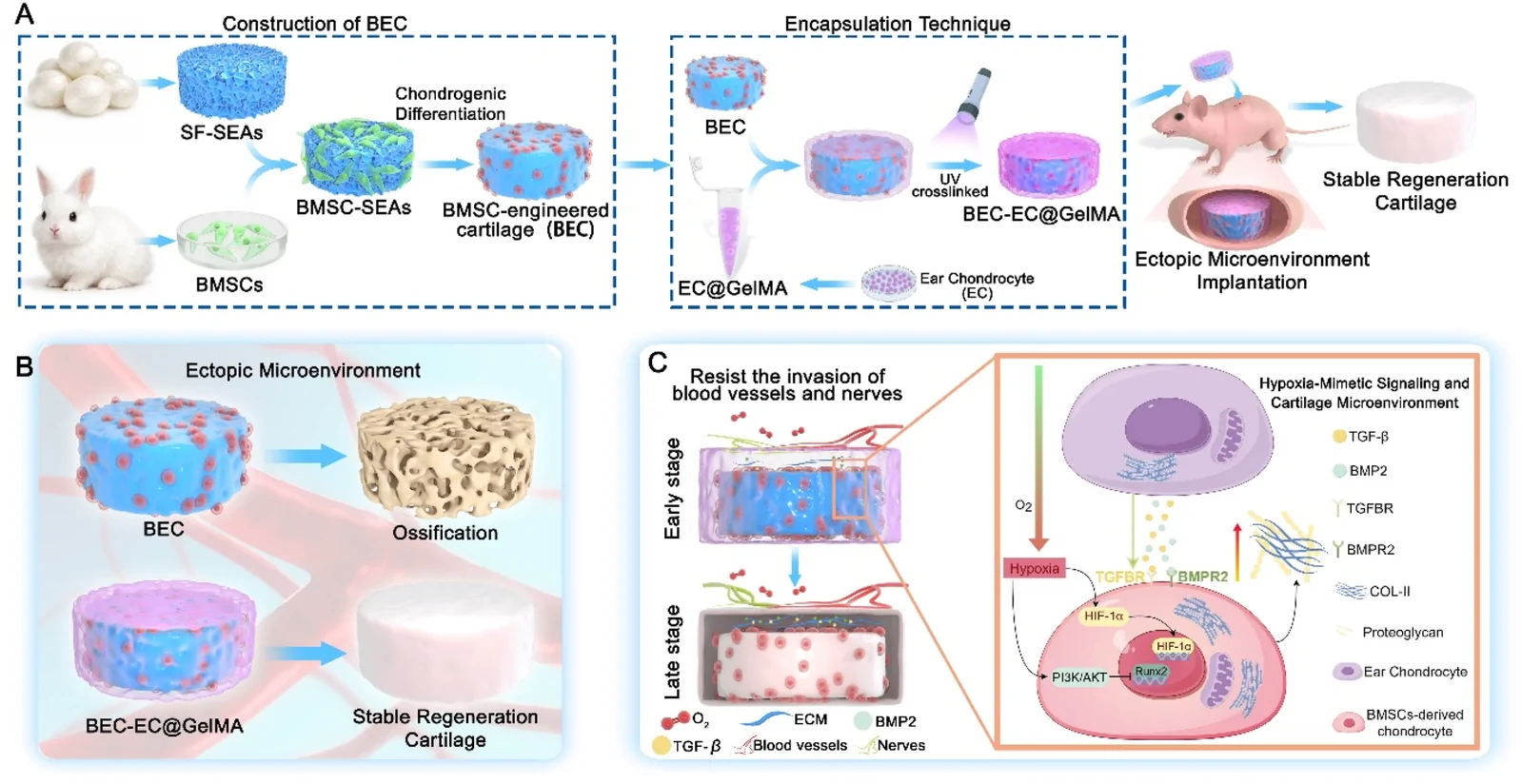
**Schematic illustration of the living cell-based encapsulation system for stable cartilage regeneration.** A) Design, preparation, and application of the BEC-EC@GelMA system. B) Results showing inhibition of endochondral ossification after ectopic implantation. C) Proposed *in vivo* mechanism by which the system inhibits ossification and promotes stable cartilage regeneration.

## 2 Results

### 2.1 Characterization of the SF-SEAs and construction of BEC *in vitro*

Silk fibroin has been accepted by U.S. Food and Drug Administration in clinic application because of its attractive material properties such as superior biocompatibility, predictable degradability and non-inflammatory byproducts, especially in the cartilage tissue engineering [[23], [24], [25]]. As shown in Fig. 2A, we created a natural SF super elastic absorbent sponges scaffold (SF-SEAs) for cell growth, cell differentiation, and cartilage regeneration. The 5.0% (w/v) SF aqueous solution, along with a cross-linking agent, underwent β-sheet formation and cross-linking reactions in a frozen environment (Fig. 2B). The epoxide groups of the cross-linking agents can bind to the amino and hydroxyl groups on the silk fiber protein, creating a chemical cross-linking network that results in the formation of a porous scaffold.

**Fig. 2:**
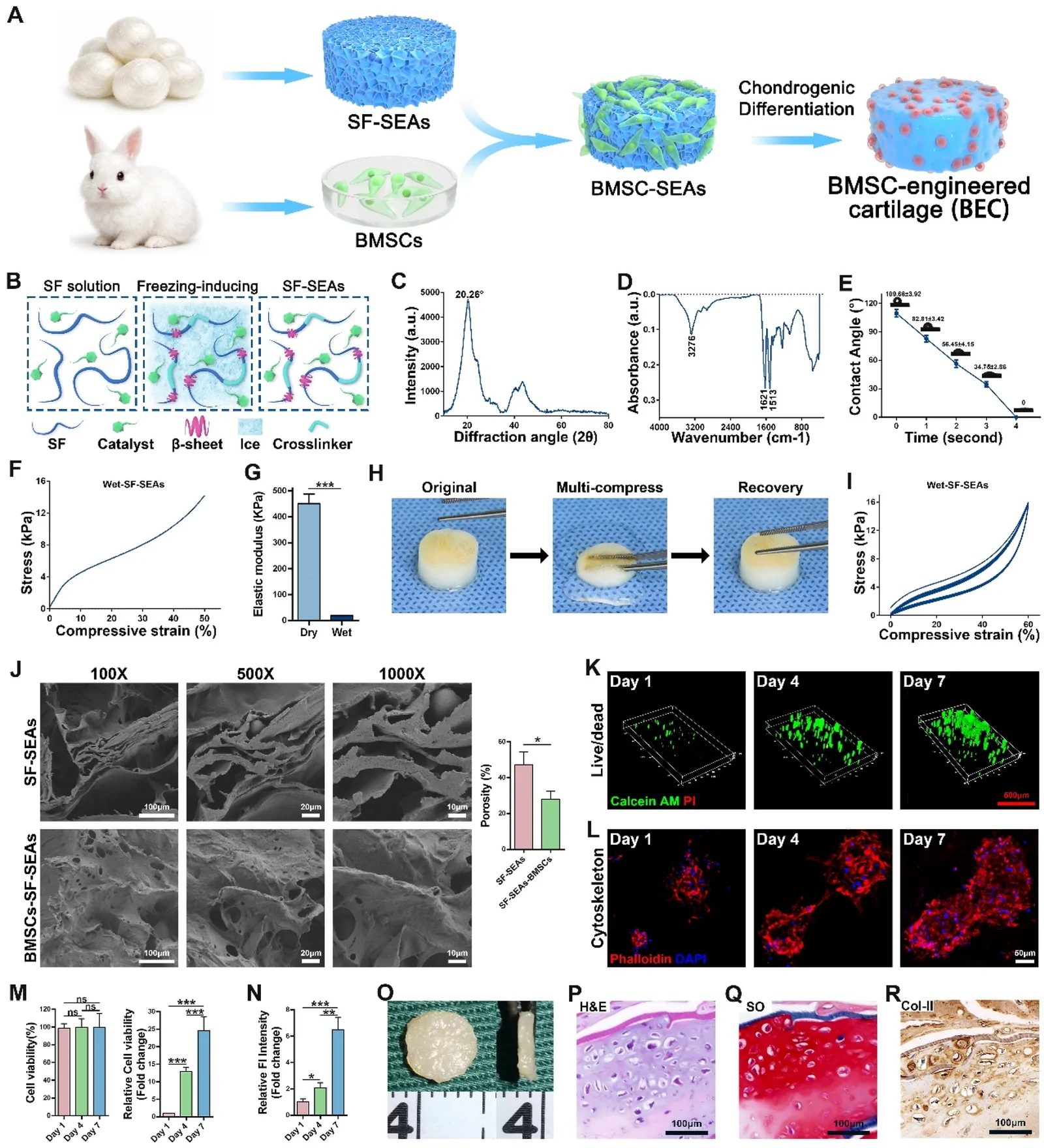
**Design, characterization of SF-SEAs, and construction of BEC *in vitro*.** A) Schematic of the BEC process. B) Mechanism of hybrid crosslinking in SF-SEAs, involving ice-templated phase separation and dual physical/chemical networks. C) XRD analysis of SF-SEAs crystallinity. D) FTIR spectra of crosslinked SF-SEAs. E) Water contact angle measurement demonstrating scaffold hydrophilicity. F) Representative compressive stress-strain curves of hydrated SF-SEAs. G) Comparison of elastic modulus between dry and hydrated scaffolds. H) Time-lapse images showing deformation and recovery of hydrated scaffolds over multiple cycles. I) Cyclic compression tests (10 cycles, 60% strain) of hydrated scaffolds. J) Surface SEM images showing morphology and porosity of acellular SF-SEAs and cell-seeded constructs (BMSC-SEAs) after 4 h of culture. Scale bars: 200 μm (100×), 20 μm (500×), 10 μm (1000×). K) Live/dead staining of BMSCs on SF-SEAs. Scale bar: 500 μm. L) Cytoskeletal staining confirming BMSC attachment and proliferation. Scale bar: 50 μm. M) Additional live/dead staining validating BMSC proliferation. N) Additional cytoskeletal staining of BMSCs. O) Gross view of the BEC after 3 weeks of chondrogenic induction. P-R) Histological staining (H&E, Safranin O, and COL II) of BECs after 3-week chondrogenic culture. Data are mean ± SD (n = 5). *, *P* ≤ 0.05, **, *P* < 0.01, ***, *P* < 0.001.

The results from the X-ray diffraction (XRD) experiments revealed a characteristic diffraction peak at 20.26° and 43.30°, indicating the typical β-sheet crystal structure of SF-SEAs (Fig. 2C). In contrast, the uncross-linked SF displayed a characteristic peak at 23.8°, which indicates the presence of an α-helical structure [26]. The fourier transform infrared (FTIR) spectrum of SF-SEAs displayed a broad absorption peak at 3276 cm^−1^, along with characteristic absorption peaks for amide I (1690-1620 cm^−1^) and amide II (1550-1510 cm^−1^). In SF-SEAs, the absorption bands for amide I and II are shifted to lower wavenumbers at 1621 and 1513 cm^−1^, respectively. This shift confirms the dual cross-linking of the cross-linker with SF (Fig. 2D). Additionally, due to its higher protein content, the SF-SEAs exhibited excellent hydrophilicity. The weight increase of the SF-SEAs after water absorption (590.90 ± 19.16 mg) was significantly greater than that of the dry SF-SEAs (48.10 ± 0.62 mg), with a maximum water absorption capacity of 1228.48 ± 39.83%. (Fig. 2E, Fig. S1A, movie S1). The SF-SEAs demonstrated markedly different mechanical properties in dry and wet states. Uniaxial compression tests were performed on SF-SEAs under both condition (Fig. 2F, Fig. S1B). The SF-SEAs exhibited higher stiffness in the dry state compared to the wet state, with elastic modulus of 451.23 ± 37.31 kPa and 19.49 ± 1.39 kPa, respectively (Fig. 2G). In the dry state, the establishment of numerous hydrogen bond interactions enhanced the stiffness of SF-SEAs, leading to a modulus approximately 23 times greater than that of wet SF-SEAs. In cyclic compression tests, wet SF-SEAs exhibited enhanced elasticity and antifatigue properties during multiple deformations (Fig. 2H–I, Fig. S1C, and movie S2). When SF-SEAs absorbed water, the hydrogen bonds between the protein's molecular chains were disrupted, weakening the interactions. As a result, the cross-linked network became the primary contributor to the mechanical properties, leading to a reduction in the modulus of SF-SEAs while enhancing their elasticity and shape recovery ability. This soft and elastic behavior closely resembles that of soft tissues.

To evaluate the cell compatibility of the cells to the scaffold, BMSCs were seeded onto the scaffolds to form BMSC-SEAs constructs and then tested by scanning electron microscopy (SEM), live/dead staining, and cytoskeletal staining. Surficial SEM images displaying the morphology features of SF-SEAs and the BMSC-SEAs constructs after 4 h of *in vitro* culture confirms that SF-SEAs had good cell adherence (Fig. 2J). Live/dead and cytoskeletal staining (Fig. 2K and L), and corresponding fluorescence ratio analysis (Fig. 2M and N) of BMSCs on SF-SEAs for 1, 4, and 7 days, which indicating stable adhesion, survival, and proliferation of the cells over time. Overall, SF-SEAs considering the superior hydrophilicity, elasticity and biocompatibility, which are beneficial for cartilage regeneration.

To evaluate the chondrogenic inducibility of the SF-SEAs, BMSC-SEAs constructs were cultured in chondrogenic induction medium (Fig. S2A–B). After three weeks of chondrogenic induction, the BMSC-SEAs constructs gradually transformed into cartilage-like tissue, which had a smooth surface, and appeared ivory-white (Fig. 2O). Histologically, the resulting BEC was characterized by the presence of typical cartilage lacunae (Fig. 2P) and cartilage-specific ECM deposition (Fig. 2Q and R).

### 2.2 EC@GelMA encapsulation technique for BEC

The BEC is prone to endochondral ossification in the ectopic microenvironment [8]. Therefore, we developed a novel technology to prevent endochondral ossification in BEC (Fig. 3A). First, the BMSCs were stained with DiO to produce green fluorescence and the ECs were stained with DiD to produce red fluorescence (Fig. 3B). Considering the superior biocompatibility and biodegradation capacity of GelMA hydrogel, thus, we constructed EC@GelMA by mixing red-dyed ECs and photo-crosslinked GelMA (EFL-GelMA-60, EFL-Tech) (Fig. S3A–C) [27], and encapsulated the BMSC-SEAs constructs with different numbers of layers to form BMSC-SEAs-EC@GelMA constructs (Fig. 3C). Hydrogels were soaked in PBS at 37 °C and reached an equilibrium swelling state within approximately 24 h (Fig. S3D). In the *in vivo* degradation study, cylindrical GelMA hydrogels retained structural integrity for up to 2 weeks after subcutaneous implantation in nude mice (Fig. S3E and F). Further *in vivo* implantation in rabbits demonstrated that both coatings remained stable on the SF-SEA scaffolds for two weeks (Fig. S3G), and the material induced a minimal local immune response while exhibiting excellent tissue compatibility throughout the observation period. As the number of encapsulation layers increased from 2 to 6, the color of the BMSC-SEAs-EC@GelMA composite progressively deepened in red (Fig. 3C). Subsequently, we used a laser confocal microscope to capture images of DiO-stained BMSC-SEAs and to examine the encapsulation effects of BMSC-SEAs within EC@GelMA with varying layers (2, 4, and 6 layers) (Fig. 3D and E). The results demonstrated that with two layers of encapsulation, a substantial number of green-stained BMSCs remained exposed. When the number of encapsulation layers was increased to 4, only a minimal number of green-stained BMSCs were detectable on the surface. Upon reaching 6 layers, the surface of the BMSC-SEAs was completely enveloped by red-stained chondrocytes. Histological examination also revealed that as the number of encapsulation layers increased, the BMSC-SEAs became more completely enclosed (Fig. 3F). We quantified the number of chondrocytes required for this encapsulation technology in different constructs of 2-layer, 4-layer, and 6-layer EC@GelMA (Fig. 3G). Live/dead staining (Fig. S4A) and the corresponding fluorescence ratio analysis (Fig. S4B) of EC@GelMA with 2, 4, and 6 layers indicated that there was no significant difference in cell viability among the three groups. Due to the limited availability of chondrocytes, traditional construction methods often involve co-cultivating chondrocytes with BMSCs to reduce the demand for chondrocytes and prevent ossification issues after the transplantation of engineered cartilage [7,18,28]. Therefore, the differences in the number of chondrocytes required in the current coating technology (4 layers) and the traditional BMSC-chondrocyte co-culture system were compared. As illustrated in Fig. S4C, the encapsulation technique necessitated a substantially lower number of chondrocytes than the traditional co-culture method. Taken together, the chondrocyte coating encapsulation system is feasible of in terms of technology and BMSC-SEAs encapsulated with 4 layers of EC@GelMA were selected for further studies.

**Fig. 3:**
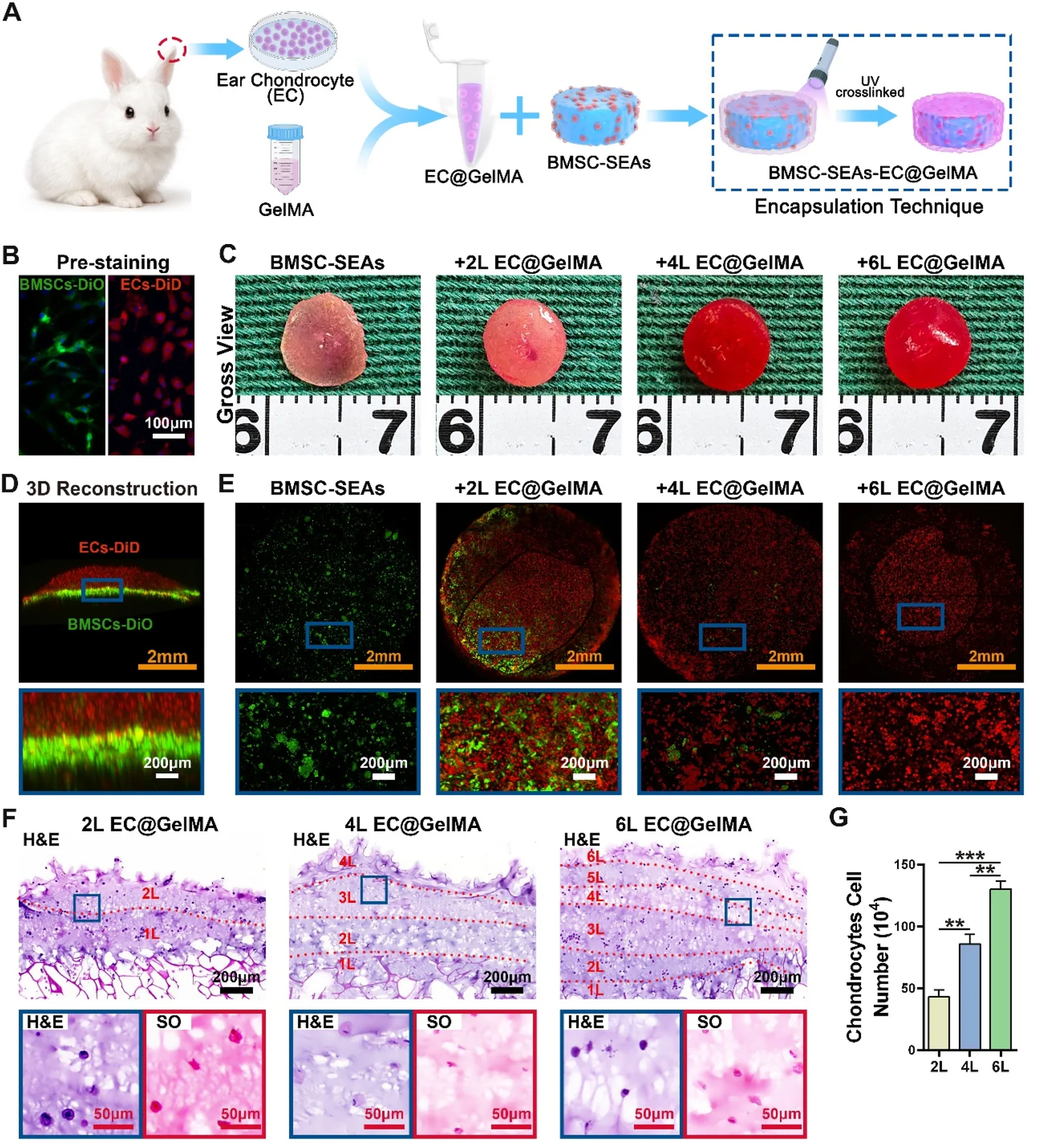
**Development and characterization of the EC@GelMA encapsulation technique.** A) Schematic of the EC@GelMA encapsulation technique. B) Fluorescent pre-staining of BMSCs (DiO, green) and ECs (DiD, red). Scale bar: 100 μm. C) Gross view of the BMSC-SEAs constructs encapsulated with 2, 4, and 6 layers of EC@GelMA. D) 3D confocal reconstruction of the BMSC-SEAs constructs encapsulated in EC@GelMA. Red scale bar: 1000 μm, white scale bar: 100 μm. E) Confocal images of BECs with varying EC@GelMA encapsulation layers. Red scale bar: 1000 μm, white scale bar: 100 μm. F) Histological staining of encapsulated BMSC-SEAs constructs with different layer numbers. Black scale bar: 200 μm, red scale bar: 25 μm. G) Quantification of ECs in 2-layer (2L), 4-layer (4L), and 6-layer (6L) EC@GelMA constructs. Abbreviations: EC, ear chondrocyte; DiO, 3,3′-dioctadecyloxacarbocyanine perchlorates; DiD, 1,1′-Dioctadecyl-3,3,3′,3′-Tetramethylindodicarbocyanine, 4-Chlorobenzenesulfonate Salt; 2L, 2 layers; 4L, 4 layers; 6L, 6 layers; H&E, hematoxylin and eosin; SO, Safranin O. Data are the mean ± SD (n = 5). **, *P* < 0.01; ***, *P* < 0.001.

### 2.3 The regulatory mechanism of EC@GelMA encapsulation technology on the internal BMSCs

To further elucidate the regulatory mechanism of the EC@GelMA encapsulation technology on the inner encapsulated BMSCs, BMSC-SEAs were cultured in regular medium for three days, followed by encapsulation with four layers of either GelMA or EC@GelMA coating. The samples were divided into three groups: the BMSC-SEAs group (control group), the BMSC-SEAs-GelMA group, and the BMSC-SEAs-EC@GelMA group. After continued cultivation in regular medium for 1-week, eukaryotic parameter transcriptome sequencing was performed. After FPKM normalization, density plots showed that the distributions of expression levels across samples were highly consistent, confirming their suitability for subsequent analysis (Fig. S5A). Box plots of gene expression for each sample revealed nearly aligned median lines, indicating successful data normalization and ensuring analytical reliability (Fig. S5B).

Principal component analysis (PCA) of the transcriptomic data showed distinct clustering of the three groups (BMSC-SEAs, BMSC-SEAs-GelMA, and BMSC-SEAs-EC@GelMA), pointing to major differences in their biological states (Fig. 4A). Volcano plots were used to visualize differentially expressed genes (DEGs) between specific comparisons: BMSC-SEAs versus BMSC-SEAs -EC@GelMA (Fig. 4B) and BMSC-SEAs -GelMA versus BMSC-SEAs-EC@GelMA (Fig. 4C). The intersection of DEGs from these two comparisons yielded a common set of 1017 genes, as illustrated in a Venn diagram (Fig. 4D). A heatmap was generated to display the expression patterns of these common DEGs across the experimental conditions (Fig. S6A).

**Fig. 4:**
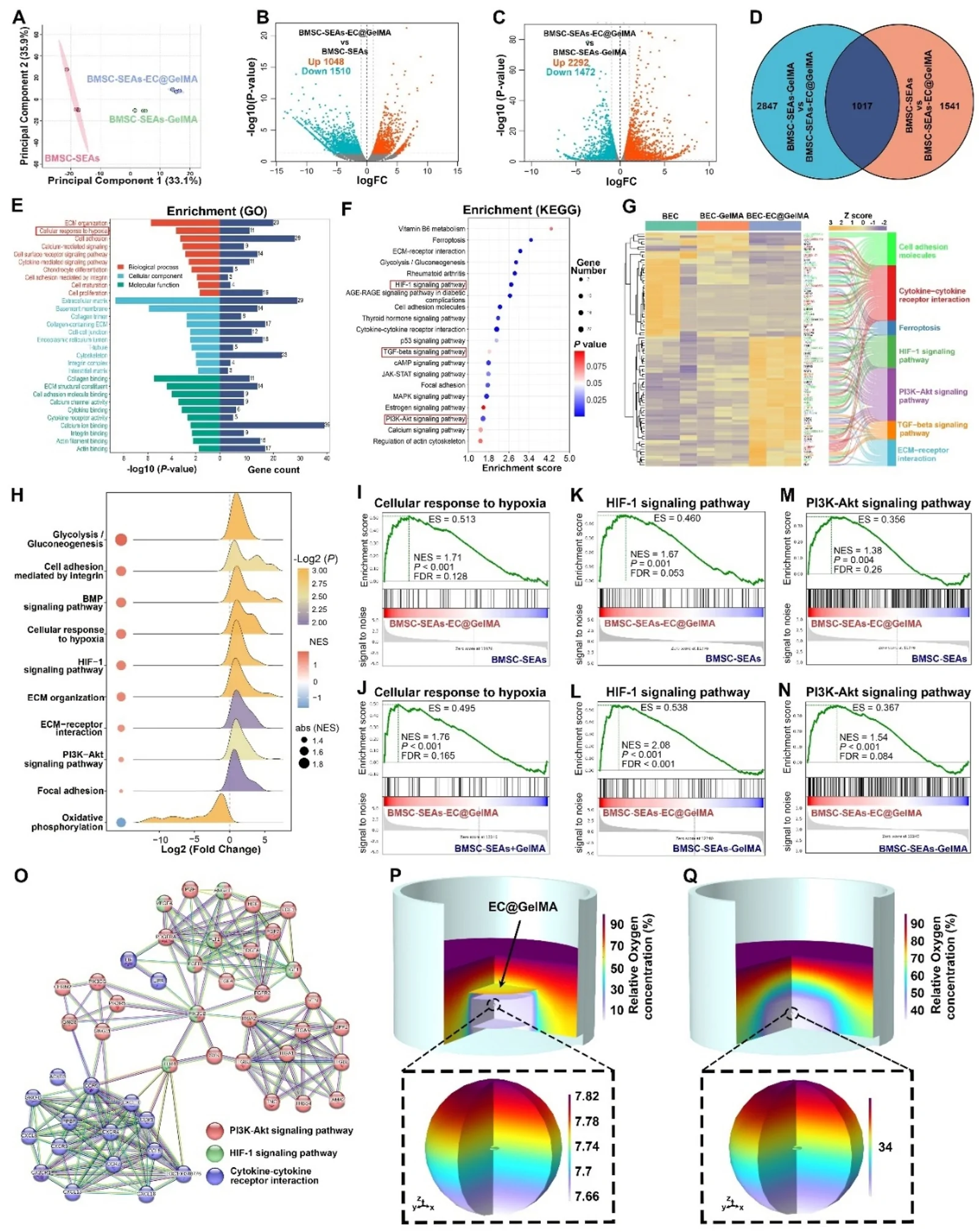
**The regulatory mechanism of EC@GelMA encapsulation technology on the encapsulated BMSCs.** A) PCA plot showing sample clustering and biological replication. B, C) Volcano plots depicting DEGs in the comparison between BMSC-SEAs-EC@GelMA and BMSC-SEAs, and between BMSC-SEAs-EC@GelMA and BMSC-SEAs-GelMA. D) Venn diagram of the intersection of 1017 common DEGs. E, F) GO and KEGG pathway enrichment analysis of the intersected DEGs, highlighting terms related to extracellular matrix and hypoxia response. G) A heatmap of genes from the major pathways associated with differentially expressed genes in BMSC-SEAs, BMSC-SEAs-GelMA, and BMSC-SEAs-EC@GelMA, including the HIF-1 signaling pathway, PI3K-Akt signaling pathway, Ferroptosis, Cell adhesion molecules, TGF-beta signaling pathway, Cytokine-cytokine receptor interaction, and ECM-receptor interaction. H) A ridgeline plot of significantly enriched GSEA pathways. I, J) GSEA pathway enrichment: cellular response to hypoxia. K, L) GSEA pathway enrichment: HIF-1 signaling pathway. M, N) GSEA pathway enrichment: PI3K-Akt signaling pathway. O) A protein–protein functional interaction network diagram of the HIF-1, PI3K-Akt, and cytokine-cytokine receptor pathways. P, Q) 3D COMSOL simulation of oxygen distribution and the oxygen concentration at the construct center for the BMSC-SEAs construct with or without EC@GelMA encapsulation.

Subsequent Gene Ontology (GO) enrichment analysis (Fig. 4E) and Kyoto Encyclopedia of Genes and Genomes (KEGG) pathway analysis (Fig. 4F) revealed that the DEGs were significantly enriched in biological processes and pathways related to extracellular matrix (ECM) organization and hypoxia response. A heatmap of genes from the major pathways associated with differentially expressed genes in BMSC-SEAs, BMSC-SEAs-GelMA, and BMSC-SEAs-EC@GelMA, including the HIF-1 signaling pathway, PI3K-Akt signaling pathway, Ferroptosis, Cell adhesion molecules, TGF-beta signaling pathway, Cytokine-cytokine receptor interaction, and ECM-receptor interaction (Fig. 4G). Consistent with this, Gene Set Enrichment Analysis (GSEA) plot analysis also showed that the pathways associated with hypoxic microenvironment were significantly enriched in the BMSC-SEAs-EC@GelMA group (Fig. 4H–N). Significantly enriched terms included cellular response to hypoxia, HIF-1 signaling pathway, PI3K-Akt, and MAPK signaling pathways. GSEA confirmed significant enrichment of both cellular response to hypoxia (BMSC-SEAs-EC@GelMA vs. BMSC-SEAs: NES = 1.71, *P* < 0.001; BMSC-SEAs-EC@GelMA vs. BMSC-SEAs-GelMA: NES = 1.76, *P* < 0.001); HIF-1 signaling pathway (BMSC-SEAs-EC@GelMA vs. BMSC-SEAs: NES = 1.67, *P* < 0.001; BMSC-SEAs-EC@GelMA vs. BMSC-SEAs-GelMA: NES = 2.08, *P* < 0.001) in the BMSC-SEAs-EC@GelMA group (Fig. 4I–L). Furthermore, signaling pathways associated with a hypoxic microenvironment (PI3K-Akt, BMSC-SEAs-EC@GelMA vs. BMSC-SEAs: NES = 1.38, *P* = 0.004; BMSC-SEAs-EC@GelMA vs. BMSC-SEAs-GelMA: NES = 1.54, *P* < 0.001, Fig. 4M and N; MAPK; Fig. S7A and B) and those crucial for cartilage biology (such as TGF-β, BMP, and ECM-receptor interaction; Fig. S7C–H) were significantly upregulated in the BMSC-SEAs-EC@GelMA group compared to both control groups. Protein-protein interaction network (PPI) network of 48 DEGs in the HIF-1, PI3K-Akt and cytokine-cytokine receptor interaction signaling pathway (Fig. 4O). To confirm the activation of the HIF-1 pathway, the protein level analysis (immunofluorescence staining) of HIF-1α and its downstream marker (VEGF) were conducted on the three groups of engineered constructs before subcutaneous implantation. As shown in Figure S7 I-L, the immunofluorescence results indicated that the proportion of HIF-1α nuclear-positive cells in the cartilage tissue of the grafts in the BMSC-SEAs-EC@GelMA group was significantly higher than that in the control group (BEC and BEC-GelMA groups).

Cartilage functional recovery is related to the hypoxic microenvironment created by high-density cell aggregates [29], as confirmed by 3D model simulation of oxygen distribution. The illustrative model reflects the steady-state distribution of oxygen throughout the BMSC-SEAs construct with or without EC@GelMA encapsulation (Fig. 4P and Q). Oxygen concentration gradually decreases from the surface to the interior region due to high-density matrix blocking and cellular consumption. Moreover, the internal oxygen concentration in the BMSC-SEAs construct without EC@GelMA encapsulation is approximately 4 Times higher than that in the construct with EC@GelMA encapsulation.

In summary, the EC@GelMA encapsulation technique can create a bioactive shield for the inner encapsulated BMSCs by integrating a hypoxic physical barrier and a biomimetic cartilage microenvironment, aiming to achieving stable chondrogenesis of BMSCs in the ectopic microenvironment *in vivo*.

### 2.4 Cartilage regeneration stability of BMSCs in the subcutaneous ectopic microenvironment

Subsequently, to evaluate whether the EC@GelMA encapsulation technology could truly inhibit the endochondral ossification of BMSCs and achieve stable cartilage regeneration in the ectopic microenvironment *in vivo*, we implanted three groups of samples subcutaneously into the dorsum of nude mice for 4 and 8 weeks (Fig. 5A). These groups included BEC (control group) after three weeks of chondrogenic induction *in vitro*, as well as BEC encapsulated in GelMA (BEC-GelMA) or EC@GelMA (BEC-EC@GelMA).

**Fig. 5:**
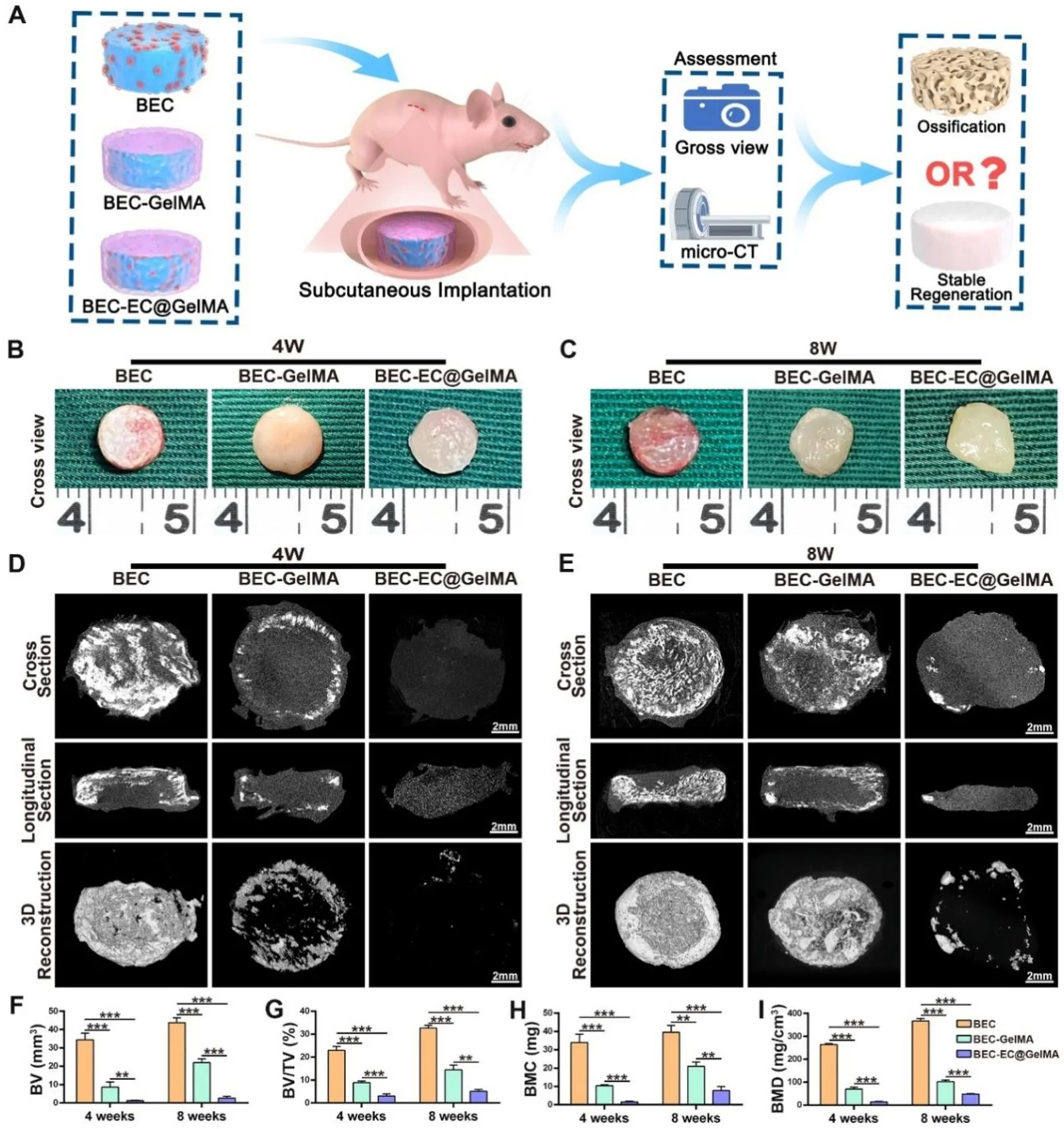
**Gross and micro-CT analysis of subcutaneous regenerated tissues of BMSCs.** A) Schematic of the subcutaneous implantation model. The schematic illustration was created with the assistance of Figdraw (https://www.figdraw.com). B, C) Gross views of explants from BEC, BEC-GelMA, and BEC-EC@GelMA groups at 4- and 8-weeks post-implantation. D, E) Representative micro-CT images (cross-section, longitudinal section, and 3D reconstruction) of explants at 4 and 8 weeks. Scale bar: 2 mm. F-I) Quantitative analysis of BV, BV/TV, BMC, and BMD. Abbreviations: BV, bone volume; BV/TV, bone volume fraction; BMC, bone mineral content; BMD, bone mineral density. **, *P* < 0.01; ***, *P* < 0.001.

As shown in Fig. 5B, the BEC group exhibited reddish bone-like tissue after 4 weeks of subcutaneous implantation. The BEC-EC@GelMA group formed smooth, ivory-white cartilage-like tissue, whereas the BEC-GelMA group displayed a white, translucent cartilage-like appearance on the surface with a reddish osseous structure internally. After 8 weeks of subcutaneous implantation, all three groups exhibited results similar to those observed at 4 weeks (Fig. 5C). Micro-CT results further confirmed that ossification occurred only in the BEC group and the BEC-gelma group, and no ossification was observed in the BEC-EC@GelMA group at 4 weeks (Fig. 5D). Meanwhile, as the subcutaneous culture time increased from 4 weeks to 8 weeks, the BEC-EC@GelMA group still showed no significant ossification (Fig. 5E). In contrast, compared with the 4-week time point, both the BEC group and the BEC-GelMA group exhibited increased ossification, with the BEC group again demonstrating the most pronounced ossification. The above results were further confirmed through the quantitative analysis of BV (Fig. 5F), BV/TV (Fig. 5G), BMC (Fig. 5H) and BMD (Fig. 5I).

Histological analysis revealed that at 4 weeks, heterogeneous structures were presented in the marginal and central regions of the BEC group and the BEC-GelMA group, including a mixture of cartilaginous tissue, bone-like tissue and connective tissue. Specifically, the cartilaginous areas displayed chondrocyte lacunae structures and deposition of cartilage ECM, as evidenced by positive staining of SO, positive expression of COLII, and blue staining with Masson's trichrome; the bone-like tissue showed positive staining of Fast Green and appeared red with Masson's trichrome (Fig. 6A–D). Furthermore, consistent with the micro-CT results, the BEC group exhibited higher levels of OCN expression and a more intensely red staining pattern in Masson's trichrome in the regenerated bone tissue compared to the BEC-GelMA group (Fig. 6E). In contrast, the BEC-EC@GelMA group (at 4 weeks) displayed mature cartilage tissue in both the peripheral and central regions, characterized by typical chondrocyte morphology and cartilage ECM deposition, while no obvious bone tissue formation was detected. Semi-quantitative analysis revealed that regarding the regenerated cartilage area and quality, the BEC-EC@GelMA group was superior to the BEC-GelMA group, which in turn was superior to the BEC group. Conversely, for the endochondral ossification area, the BEC-EC@GelMA group was lower than the BEC-GelMA group, which was also lower than the BEC group (Fig. 6F–J).

**Fig. 6:**
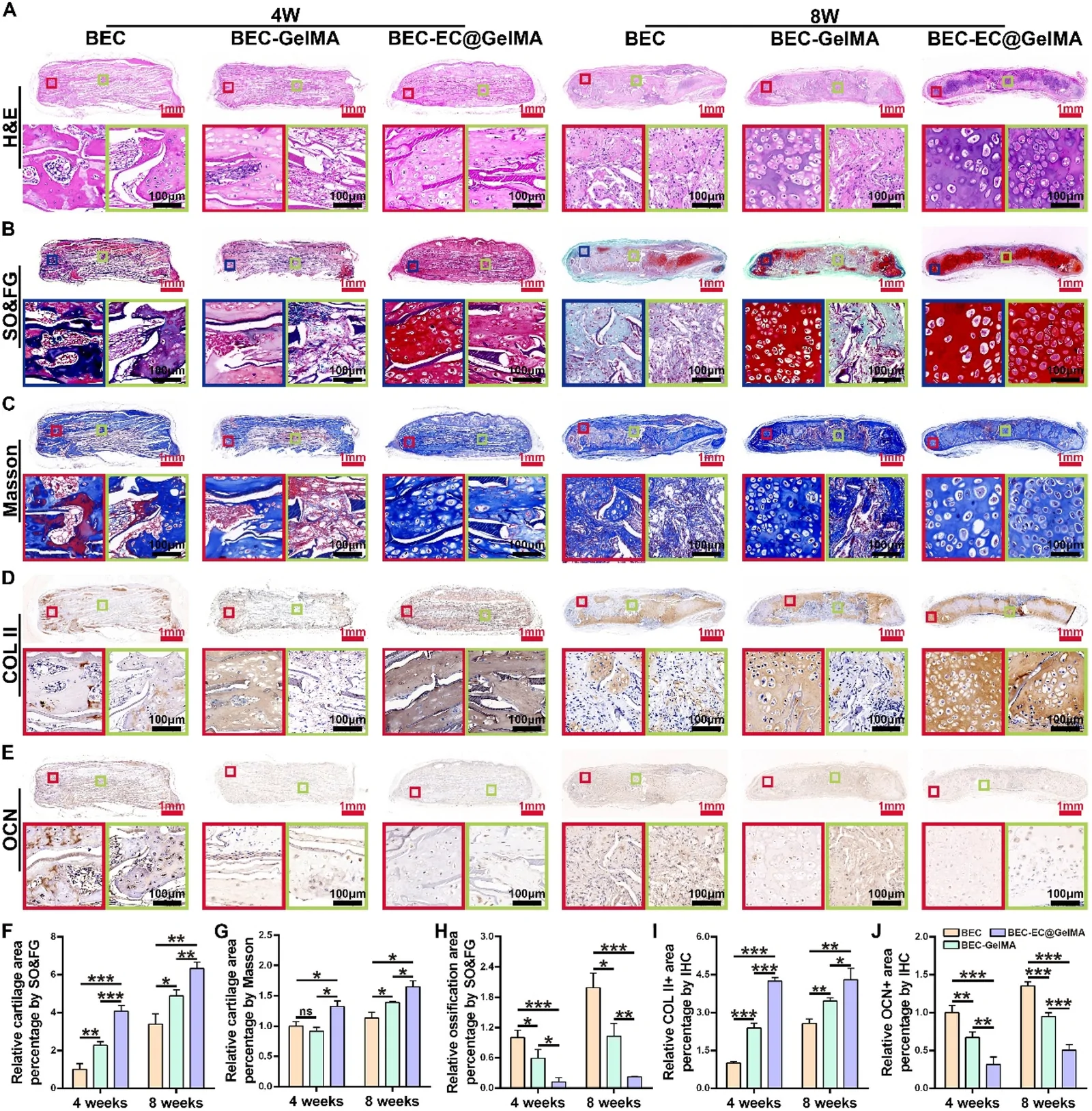
**Histological analysis of subcutaneous regenerated tissues of BMSCs.** A-E) Histological staining (H&E, SO&FG, Masson's trichrome, COL II, and OCN) of explants from BEC, BEC-GelMA, and BEC-EC@GelMA groups at 4- and 8-weeks post-implantation. Red scale bar: 1 mm, black scale bar: 100 μm. F, G) Relative regeneration cartilage area percentage by SO&FG and Masson's trichrome. H) Relative ossification area percentage by SO&FG. I, J) Relative COL II+ and OCN + area percentage by IHC. The red border represents the central region, and the green border represents the peripheral region. Abbreviations: H&E, hematoxylin and eosin; SO&FG, Safranin O and Fast Green. **, *P* < 0.01; ***, *P* < 0.001.

As the subcutaneous implantation time was extended to 8 weeks, the histological results of the three groups were consistent with the observations at 4 weeks, with the regenerated bone tissue exhibiting more mature in the BEC and BEC-GelMA group. Briefly, the peripheral and central regions of the BEC group were characterized by the presence of bone-like tissue, as confirmed by the positive expression of bone-related ECM. The edge of the BEC-GelMA group continued to present cartilage morphology, whereas the central area showed obvious presence of bone-like tissue and connective tissue. In contrast, the entire region of the BEC-EC@GelMA group formed more mature and homogeneous cartilage tissue throughout the entire region compared to that after 4 weeks of subcutaneous culture, characterized by more typical cartilage lacunae and more abundant cartilage ECM deposition.

We further evaluated the biosafety of BEC, BEC-@GelMA, and BEC-EC@GelMA using H&E-stained histological images. The cells in the heart, liver, spleen, lung, and kidney from all three groups exhibited normal morphology with no abnormal histological changes compared to the control group (Fig. S8A). These results indicate that EC@GelMA encapsulation offers high biosafety and good biocompatibility *in vivo*.

### 2.5 Anti-angiogenic and anti-neurogenic evaluation

Previous studies have confirmed that the presence of blood vessels and nerves is a crucial factor in directing the osteogenic differentiation of bone marrow mesenchymal stem cells and the subsequent regeneration of bone tissue [13,14]. Therefore, we further performed histological analyses to assess angiogenesis and neurogenesis in BMSC-regenerated tissues harvested from the subcutaneous dorsum of nude mice at 4- and 8-weeks post-implantation. First, immunofluorescence staining for CD31 and α-smooth muscle actin (α-SMA) revealed that among the BMSC-regenerated tissues cultured subcutaneously for 4 weeks and 8 weeks, the BEC group exhibited the highest level of vascular expression, whereas the BEC-EC@GelMA group showed the lowest (Fig. 7A–D, Fig. S9A and B). Additionally, we performed immunofluorescence staining for protein gene product 9.5 (PGP9.5) to qualitatively and quantitatively evaluate the distribution of nerve fibers. The results showed that at both 4 and 8 weeks, the BEC group displayed the highest number of nerve fibers, a small number of nerve fibers were distributed in the BEC-GelMA group, while the BEC-EC@GelMA group showed negative PGP9.5 staining (Fig. 7E–H, Fig. S9C and D). Quantitative analysis revealed that the number of blood vessels (Fig. 7C and D) and nerve fibers (Fig. 7G and H) decreased when BECs were encapsulated with GelMA or EC@GelMA. As the duration of implantation in the subcutaneous ectopic microenvironment increased, the SF-SEAs degraded progressively, concomitant with further maturation of the cartilage tissue and a reduction in blood vessel and nerve invasion into the BEC, BEC-GelMA, and BEC-EC@GelMA constructs.

**Fig. 7:**
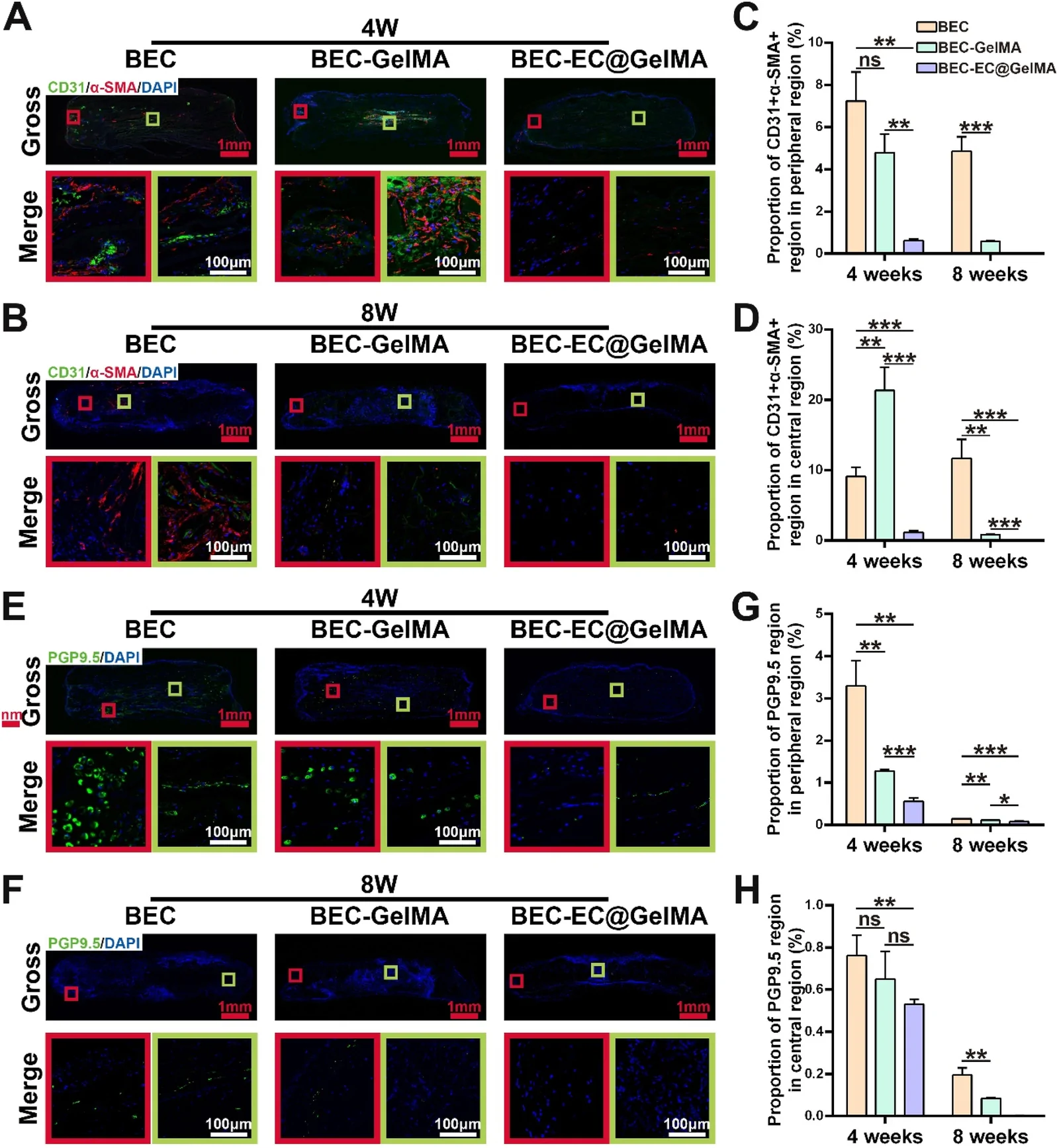
**Immunofluorescence analysis of neurovascularized invasion inhibition.** A, B) Double immunofluorescence staining for CD31 (green) and α-SMA (red). Nuclei are counterstained with DAPI (blue) C, D) Quantitative analysis of the α-SMA+/CD31+ area in central and peripheral region of BEC, BEC-GelMA, and BEC-EC@GelMA groups. E, F) Immunofluorescence staining for PGP9.5 (green). Nuclei are counterstained with DAPI (blue). G, H) Quantitative analysis of the PGP9.5+ area in central and peripheral region of BEC, BEC-GelMA, and BEC-EC@GelMA groups. The red border represents the central region, and the green border represents the peripheral region. n.s., *P* > 0.05; *, *P* ≤ 0.05; **, *P* < 0.01; ***, *P* < 0.001. Red scale bar: 1 mm, white scale bar: 100 μm.

These results further confirmed that GelMA acted as a physical barrier, partially inhibiting the invasion of blood vessels and nerve fibers, while EC could enhance this functionality. The presence of blood vessels and nerve fibers was observed exclusively in the ossification area, highlighting the absence of these structures in the cartilage region.

## 3 Discussion

The repair of cartilage injuries is a major challenge in the field of regenerative medicine [30]. Cartilage regeneration utilizing BMSCs and BMSCs-derived exosomes had become a promising therapeutic approach for repairing cartilage defects [31,32]. However, upon implantation into an ectopic microenvironment (such as the subcutaneous microenvironment), BMSCs are highly prone to ossification, leading to failure of cartilage regeneration [33]. Consequently, BEC has not yet achieved widespread clinical translation. To overcome this limitation, in the current research, we innovatively developed a living cell-based encapsulation coating technique. Hydrogels have been widely used in tissue bioengineering, drug delivery, wearable devices, and cell-based therapy [[34], [35], [36], [37]]. Specifically, the surface of BECs was coated and encapsulated with EC@GelMA, formed by chondrocytes and GelMA, prior to implantation into an ectopic *in vivo* microenvironment, with the aim of creating a biophysical barrier to maintain the stability of BMSCs regenerated cartilage in an ectopic microenvironment. The results of *in vitro* experiments confirmed that, technically, EC@GelMA can encapsulate the internal BMSCs, and the number of chondrocytes required is significantly reduced compared with the conventional BMSC-chondrocyte co-culture techniques; Theoretically, transcriptome sequencing results show that EC@GelMA can provide a hypoxic and bioactive cartilage-mimetic microenvironment for the internally encapsulated BMSCs. Ultimately, *in vivo* experiments demonstrated that in the subcutaneous ectopic microenvironment, the EC@GelMA coating technique could maintain the stability of cartilage regeneration of BMSCs and gradually form mature cartilage tissue. These findings suggest that the EC@GelMA encapsulation system establishes a bionic, multi-layered protection system - featuring both hypoxia and biological barriers - which synergistically combines immediate physical shielding with long-term biological regulation. In conclusion, the chondrocyte encapsulation coating technology can provide BMSCs with a biomimetic cartilage microenvironment that isolates blood vessels and nerves, enabling stable cartilage regeneration of BMSCs in an ectopic microenvironment.

A suitable scaffold vector for BMSCs is a prerequisite for verifying whether the cell coating technology can maintain the stability of cartilage regeneration of BMSCs in the ectopic microenvironment. Currently, many types of synthetic and natural scaffolds are used for cartilage engineered regeneration [38]. As natural scaffolds, SF-SEAs not only exhibit fundamental properties such as excellent biocompatibility, but also notably, its excellent hydrophilicity and elasticity have recently drawn significant attention in the field of cartilage tissue engineering [21,39]. Based on previously established methods, as shown in Fig. 2, SF-SEAs were successfully prepared. Consistent with the results of previous studies, it was confirmed that SF-SEAs have excellent mechanical properties, cell adhesion properties and cell compatibility. Furthermore, BMSC-SEAs were constructed and gradually transformed into cartilage-like tissue after three weeks of chondrogenic induction (Fig. 2O–R). Taken altogether, the above-mentioned characteristics make SF-SEAs subsequent research on chondrocyte coating encapsulation technology an ideal scaffold material for maintaining the stability of cartilage regeneration of BMSCs in ectopic microenvironments.

After the BEC was constructed, we primarily aimed to develop how to maintain the stability of BMSC-regenerated cartilage in the ectopic microenvironment. To mitigate ectopic ossification in BMSC-based cartilage regeneration, two principal strategies have been explored: mixed co-culture with chondrocytes and encapsulation within anti-angiogenic drug materials [7,19,40,41]. While co-culture takes advantage of chondrogenic paracrine signaling, its effectiveness is limited by unpredictable cell interactions, inconsistent differentiation outcomes, and a heavy reliance on scarce chondrocytes, posing major challenges for clinical translation. Conversely, electrospun membranes provide a localized pharmacological barrier but function passively, lacking the capacity to actively direct chondrogenesis and facing risks associated with drug toxicity, unsustainable release kinetics, and finite duration of action. Therefore, in this study, to overcome these constraints, we developed the chondrocyte coating encapsulation technology to provide sustained chondrogenic instruction and a physiologically active barrier within a single, scalable construct. Briefly, as shown in Fig. 3,red-dyed ECs and photo-crosslinked GelMA were mixed to form EC@GelMA, which was then coated onto the surface of green-stained BMSC-SF-SEA composites with different numbers of layers. Confocal and histological staining results confirmed that this coating technique could encapsulate BMSC-SEAs, and the encapsulation effect became better as the number of coating layers gradually increased from 2 to 6 (Fig. 3D–F). Meanwhile, in order to reduce the number of chondrocytes used, the number of coating layers was set to 4 (Fig. 3G). Collectively, the original intention behind designing this chondrocyte coating technology is based on the following theoretical considerations, 1) the chondrocyte coating technology functions through an early-stage physical barrier primarily formed by the hydrogel [42,43], and a mid-to-late-stage biophysical barrier established as the hydrogel gradually degrades while the chondrocyte coating progressively develops into cartilage tissue; 2) Meanwhile, the chondrocyte (cartilage tissue) coating continuously provides ECM and cytokines that induce chondrogenesis [44,45]. Moreover, due to the gradual maturation of peripheral cartilage tissue, it further promotes the chondrogenic differentiation of internal BMSCs and enables the stable cartilage regeneration. 3) Furthermore, as a natural cellular barrier, it offers a more physiologically, multifunctional, and adaptive shielding system. The coating cells can potentially regulate their barrier properties and secretory characteristics in response to local environmental changes, achieving dynamic and benign feedback regulation.

After constructing the chondrocyte coating model, the next step is to verify the regulatory effect of this coating encapsulation technology on the inner BMSCs and its underlying mechanisms. We performed eukaryotic parametric transcriptome sequencing on the three groups: BMSC-SEAs (control group), BMSC-SEAs-GelMA and BMSC-SEAs-EC@GelMA group. Transcriptomic sequencing elucidated the mechanism: GO and KEGG analyses indicated significant enrichment of differentially expressed genes in “cellular response to hypoxia” and the “HIF-1 signaling pathway” in the BMSC-SEAs-EC@GelMA group (Fig. 4D and E). GSEA confirmed marked activation of these pathways (Fig. 4F–K). Additionally, 3D COMSOL simulation data show that EC@GelMA technology can provide a hypoxic microenvironment for the internal BMSCs (Fig. 4P and Q). Furthermore, before subcutaneous implantation, the protein levels of HIF-1α and its downstream index (VEGF) in the three groups of engineered constructs were detected and analyzed, and the results confirmed that the proportion of HIF-1α nuclear-positive cells in the cartilage tissue of the graft within the BMSC-SEAs-EC@GelMA group was significantly higher than that in the other two groups (BEC group and BEC-gelma group) (Fig. S7 I-L). These findings indicate that ECs within GelMA recapitulate and augment the hypoxic microenvironment typical of native articular cartilage. Under these conditions, HIF-1α acts as a central regulator. It upregulates chondrogenic genes like *SOX9* and *COL2A1* to boost cartilage-specific matrix production and sustain a synthetic cell state, while simultaneously inhibiting pro-angiogenic factors such as VEGF, thereby biochemically preventing vascular invasion. [[46], [47], [48], [49]]. Thus, ECs act as active biosynthetic units that secrete paracrine factors (e.g., TGF-β, BMP2) to sustain cartilage homeostasis and counteract ossification, even as the GelMA barrier degrades [50,51]. This dual mechanism hinges on initial physical isolation followed by sustained biological regulation, offering an innovative and effective way to prevent engineered cartilage from ossifying *in vivo*.

Although we have confirmed the feasibility of the current chondrocyte coating encapsulation technology in terms of theory, technology and mechanism, to determine whether this technology can truly inhibit the endochondral osteogenesis of BMSCs in the ectopic microenvironment *in vivo* and achieve stable cartilage regeneration, *in vivo* experiments are ultimately needed for verification. Therefore, we implanted BEC (control group), BEC-GelMA and BEC-EC@GelMA group into subcutaneous microenvironments on the backs of nude mice for 4-weeks and 8-weeks, respectively. The results from gross observation, micro-CT, and histological assessments of chondrogenesis and osteogenesis confirmed that ossification occurred in the BEC and the BEC-GelMA groups, with the BEC group exhibiting more severe ossification (Fig. 5B–H). In contrast, no ossification was observed in the BEC-EC@GelMA group at 4 weeks, and instead, cartilage tissue with chondrocyte lacunae and deposition of cartilage extracellular matrix was formed. As shown in Fig. 5, when the subcutaneous culture period was extended to 8 weeks, the BEC-EC@GelMA group still showed no significant ossification and developed more mature cartilage tissue. In contrast, both the BEC and BEC-GelMA groups displayed increased ossification compared to the 4-week time point, with the BEC group again showing the most pronounced ossification. Furthermore, previous studies have confirmed that blood vessels [52,53] and nerves [54]in the ectopic microenvironment are the major factors leading to endochondral ossification of BMSCs. Therefore, further, through qualitative and quantitative detection of specific markers of blood vessels and nerves in the subcutaneous regenerated tissues, it was confirmed that blood vessels and nerve fibers were only observed in the ossification area, while these structures were not absent in the cartilage area. In summary, chondrocyte coating encapsulation technology is not only theoretically and technically feasible, the above research results confirm that as a functional biological barrier, in the challenging *in vivo* environment, it physically blocks blood vessels and the nervous system from the host's ectopic microenvironment and the bone-promoting components they provide. Prevent them from directly approaching and “misleading” the encapsulated BMSCs, thereby inhibiting endochondral ossification during the cartilage regeneration process of BMSCs in the subcutaneous ectopic microenvironment and ultimately achieving stable cartilage regeneration. This active isolation represents a critical feature that previous methods such as co-culture of chondrocytes with BMSCs or anti-vascular drug delivery strategies cannot provide.

Although this study has achieved positive results, as an exploratory work, it still has several limitations, which also point the way for future research. Firstly, there is a lack of studies on BMSC chondrogenesis in ectopic microenvironments using large animal models, as well as investigations into long-term stability (for example, subcutaneous ectopic microenvironment culture for half a year or even longer). Secondly, considerable work remains regarding the depth of mechanism and the translational pathway. Future research will focus on: exploring optimized and programmable coating techniques, such as DLP inkjet encapsulation technology, to achieve more precise, reproucible, and scalable fabrication processes; mechanistic dissection via proteomic profiling of EC secretomes, coupled with functional rescue assays using inhibitors or agonists to pinpoint effector molecules, conducting experimental validations in large-animal models and comprehensive preclinical evaluations of biosafety to facilitate eventual clinical translation. Thirdly, the model lacks the native mechanical load, vascularity, and immune microenvironment characteristic of craniofacial cartilage; furthermore, the inert subcutaneous environment may not reliably predict coating performance under dynamic compression or in the presence of host immune cells.

## 4 Conclusion

This study addresses the clinical challenge of ectopic ossification in BMSC-based engineered cartilage by innovatively developing a chondrocyte-GelMA encapsulation coating technique (EC@GelMA). This strategy establishes a hypoxic, biomimetic microenvironment for the encapsulated BMSCs and creates a dynamic dual barrier system (physical and biological). It successfully inhibited neurovascularized invasion as well as ossification in a subcutaneous ectopic model, achieving stable and long-term cartilage regeneration from BMSCs. This work provides a novel and effective “material-cell” synergistic strategy to overcome a key bottleneck in the clinical translation of tissue-engineered cartilage.

## 5 Materials and methods

### 5.1 Preparation of the SF-SEAs scaffold

Building on our previous research findings, we have developed the SF-SEAs scaffold suitable for cartilage regeneration [21]. Briefly, insoluble scaffolds of SF-SEAs were fabricated with SF solution, ethylene glycol diglycidyl ether (EGDE, CAS 26403-72-5, Macklin, China) and N,N,N′,N′-tetramethylethylenediamine (TEMED, CAS 110-18-9, Macklin, China). SF aqueous solution (5.0% w/v) was mixed with EGDE (3 mmol/g) and TEMED (0.25 v/v %) with continuous stirring in an ice bath. Then, the mixture was transferred into molds of cylinder, first for rapid freeze-setting at − 80 °C for 1 h, followed by a cryogen reaction at − 10 °C for 1 day, and then thawed at room temperature for 6 h. The reacted insoluble scaffolds were rinsed in ultrapure water to remove residual crosslinker and catalyst. Finally, the SF-SEAs were freeze-dried in vacuum.

### 5.2 Scanning electron microscopy

Sections of the SF-SEAs microstructures were observed with scanning electron microscopy (Zeiss Gemini 300, Germany) at an accelerating voltage of 15 kV. All samples were sputter-coated with gold before observation.

### 5.3 Fourier transform infrared spectroscopy

Attenuated total reflectance FTIR spectra of SF-SEAs were recorded with a Thermo Scientific Nicolet iS20 FTIR spectrometer (Thermo Fisher Scientific, USA) to identify the crosslinked groups of the SEAs. The FTIR spectra were recorded in the wavenumber range of 400–4000 cm^−1^ by accumulating 32 scans under a resolution of 4 cm^−1^.

### 5.4 X-ray diffraction

The crystallinity of the SF-SEAs w assessed using by a Rigaku SmartLab SE diffractometer (Rigaku, Japan) equipped with a Cu Kα radiation source (wavelength of 0.1542 nm). The Angle measurement range of this experiment is 10–80° and the scanning mode was step-scan with a step length and scanning speed of 1°/min. Prior to testing, all samples were then divided into slices that were 2 mm thick.

### 5.5 Contact angle test

The wettability characteristics of the SF-SEAs were evaluated using by SDC-350KS Contact Angle system (Sindin, China). Each sample, shaped into cylindrical forms, was placed on the measurement surface. A droplet of deionized water, approximately 2 μL, was deposited gently onto the sample surface using an automatic pipette. Images of the droplet were captured at 62 ms intervals with a high-resolution camera, allowing for the subsequent calculation of the contact angle from the static liquid deposition images. By applying the Young–Laplace equation around the droplet with the system software, the contact angle was determined.

### 5.6 Mechanical characteristics of scaffolds

The mechanical properties of the SF-SEAs were assessed using a general testing machine (Instron 5542, Canton, USA), following the methodology previously described [39]. The SF-SEAs were processed into cylindrical shapes with a diameter and thickness of 4 mm. To evaluate the properties of the wet SF-SEAs, the samples were equilibrated in phosphate-buffered saline (PBS, Gibco-BRL, USA) at room temperature prior to testing. A deformation rate of 1 mm/min was applied, compressing the SF-SEAs to a maximum strain of 50% during single axial compression tests and 60% during cyclic axial compression tests. The elastic modulus was determined from the relatively linear region of the stress-strain curves, specifically within the 20–40% strain interval [21].

### 5.7 Preparation of BMSCs and chondrocytes

In this study, all animals received humane care, in accordance with the National Institutes of Health Guide for the Care and Use of Laboratory Animals (NIH Publications No. 8023, revised 1978). All protocols for experiments in animals were approved by the Animal Care and Experimental Committee of Shanghai Rat&Mouse Biotech Co.,Ltd (220250914 (13)). All samples were radiation sterilized for subsequent tests.

As previously reported, bone marrow was harvested from the iliac bone of adult New Zealand white rabbits. The isolated BMSCs were cultured in regular culture medium composed of Dulbecco's Modified Eagle Medium (DMEM, Gibco-BRL, USA) containing 10% fetal bovine serum (FBS, Gibco-BRL) and 1% penicillin–streptomycin solution (Gibco BRL), under a controlled environment of 37 °C and 5% CO_2_ [7,16]. After 5 days, the medium was changed to remove all non-adherent cells following previously reported protocols. BMSCs were passaged using 0.25% trypsin. BMSCs at second passage were harvested for use.

Auricular cartilage was obtained from rabbits and cut into 1.0 mm^3^ pieces before being washed with PBS and digested with 0.15% collagenase NB4 (Nordmark Biochemicals, Germany) to isolate chondrocytes, according to previously established methods [7,21]. The isolated chondrocytes were cultured in DMEM containing 10% FBS and 1% penicillin–streptomycin solution. Chondrocytes at second passage were used in downstream experiments.

### 5.8 Cellular attachment and cell viability of SF-SEAs scaffolds

BMSC suspension at a concentration of 1.0 × 10^7^ cells per mL was evenly seeded onto the SF-SEAs, and the constructs were incubated for 4 h in a humidified incubator at 37 °C under an atmosphere containing 5% CO_2_. Then, the scaffolds with BMSCs were fixed overnight in 0.05% glutaraldehyde at 4 °C. After dehydration in a graded ethanol series, samples were subjected to critical-point drying, and the attachment of BMSCs on the scaffolds were observed with SEM at an accelerating voltage of 15 kV. All samples were sputter-coated with gold before observation. After 1, 4, and 7 days of culture, the proliferation activity of BMSCs on the scaffolds were determined using the live/dead staining (Invitrogen, USA) following the manufacturer's instructions. Meanwhile, the cytoskeleton was stained with phalloidin (Meilunbio, China) and counterstained with DAPI (C1006, 1 mg/mL, Beyotime, China) to directly analyze the morphological features of the BMSCs seeded onto SF-SEAs. The fluorescence of Live/dead staining and cytoskeleton was detected by using Leica TCS SP8 3X microscope (Leica, Germany). In addition, the fluorescence image data were analyzed to evaluate the cell viability and cellular attachment of scaffolds by using Fiji (ImageJ2) Version 2.9.0 (NIH, Bethesda, USA).

### 5.9 Preparation of BMSC-SEAs constructs and BEC

BEC was constructed as reported previously [21]. Briefly, a BMSC suspension (1.0 × 10^7^ cells/mL) was evenly seeded onto the SF-SEAs constructs. The constructs were first incubated in regular medium (DMEM, 10% FBS, 1% Penicillin-Streptomycin solution) for 4 h, then cultured for 3 days in a 5% CO_2_ incubator at 37 °C. Thereafter, the constructs were cultured in chondrogenic induction medium composed of DMEM, 10 ng/mL transforming growth factor-β1 (TGF-β1, R&D Systems, USA), 50 ng/mL insulin-like growth factor-I (IGF-I, R&D Systems), 1% ITS, and other defined supplements (serum-free, all components chemically defined) [16,55]. The culture medium was changed every 3 days, and histological examination of the BEC samples was performed following 3 weeks of *in vitro* culture.

### 5.10 Development strategy of the EC@GelMA encapsulation technique

After the construction of BECs, we developed the chondrocyte coating encapsulation technology that can effectively encapsulate BEC, thereby maintaining the stability of cartilage regenerated by BMSCs in an ectopic microenvironment. Briefly, P2 ear chondrocyte (EC) were mixed with GelMA (EFL-GelMA-60 5g, EFL-Tech(suzhou) co., LTD., China) to form EC@GelMA (3 × 10^7^ cells/mL). Then the EC@GelMA mixture was then coated onto the surface of the BECs. Subsequently, photopolymerization was carried out using ultraviolet light to fix EC@GelMA onto the entire surface of the BEC.

To validate the effectiveness of this technology, we conducted visualization experiments. We first stained the EC with DiD (red fluorescence, C1039-10 mg, Beyotime) and the BMSCs with DiO (green fluorescence, C1993S, Beyotime). BMSC-SEAs were constructed and EC@GelMA was prepared following the method described above, and then the BMSC-SEAs were coated with 2, 4, and 6 layers of EC@GelMA, respectively. The BMSC-SEAs encapsulated with different layers of EC@GelMA (2, 4 and 6) were observed using a laser confocal microscope to examine whether the chondrocyte coating encapsulation technology could effectively encapsulate BMSCs internally and to assess the encapsulation efficacy of different layers. Additionally, we performed H&E and SO staining on BMSC-SEAs encapsulated with 2, 4, and 6 layers of EC@GelMA to further verify the feasibility of the chondrocyte coating encapsulation technique. In selecting the optimal encapsulation thickness, we aimed for complete coverage while avoiding excessive thickness that could impede nutrient permeability. After considering these factors, we ultimately chose BMSC-SEAs encapsulated with 4 layers of EC@GelMA for subsequent experiments.

### 5.11 Transcriptome RNA-sequencing and bioinformatics analysis

Briefly, samples of different BMSC-SEAs construct groups were collected and transferred into Invitrogen TRIzol solution (n = 3 per group). Total RNA was extracted using TRIzol reagent (Invitrogen, USA), and genomic DNA was removed using DNase I (Takara, Japan). Then, total RNA was treated with Oligo(dT)-labeled magnetic beads to enrich and purify mature mRNA. RNA purity, concentration, and integrity were assessed using a NanoDrop™ One spectrophotometer (Thermo, USA) and an Agilent 2100 Bioanalyzer (Agilent, USA), respectively. A fragmentation buffer was then added to fragment the mRNAs. These fragments were reverse-transcribed into cDNA with random primers. The cDNA fragments were purified using the QIAQuick PCR kit (Qiagen, USA), end-repaired, poly(A)-tailed, and ligated to Illumina sequencing adapters. Fragments of the target size were recovered by agarose gel electrophoresis, amplified by PCR, and subjected to sequencing using an Illumina NovaSeq™ 6000 (Illumina, USA) at OE Biotech, Inc. (Shanghai, China). We acknowledge the support provided by OE Biotech, Inc. in sequencing and bioinformatics analysis. Gene expression levels were quantified as fragments per kilobase of transcript per million mapped reads (FPKM) using StringTie. Three biological replicates per group were included in the sequencing experiment.

Bioinformatics analysis was performed as previously reported [56,57]. Differential expression analysis was primarily conducted using the “DESeq2” R package on raw read counts. Normalization was performed internally by DESeq2 using the median-of-ratios method. Genes with an adjusted p-value (q-value) ≤ 0.05 and an absolute fold change > 2 (or < 0.5) were considered significantly DEGs. To confirm the robustness of the results, a separate analysis using the “voom” function (from the “limma” R package) was also performed, yielding highly concordant DEGs. Heatmaps were generated using the “pheatmap” R package, and volcano plots were displayed using the “ggplot2” R package. Based on the hypergeometric distribution, GO, KEGG, and GSEA enrichment analyses of DEGs were performed using the online tool (https://cloud.oebiotech.com).

### 5.12 COMSOL simulation of oxygen distribution

Model simulations were performed using COMSOL Multiphysics software, as previously reported [58]. To study the characteristics of the BMSE-SEAs construct with or without EC@GelMA encapsulation within this system, a mathematical model was developed by leveraging cylindrical symmetry. The oxygen concentration distribution in a petri dish containing culture medium was simulated using the Transport of Diluted Species interface. The dynamics of oxygen in BMSE-SEAs construct culture are described by the following equation:

∇·(−D∇c)=R

where *c* denotes the oxygen concentration (mol/m^3^), *D* is the diffusion coefficient (m^2^/s), and *R* represents the reaction rate (mol/m^3^/s). This equation follows Fick's second law of diffusion.

A continuous consumption reaction is applied on the surface of the construct to account for oxygen consumption by the cells:

$$R=-kc\;\cdot \;C_{O2}$$

where *k*c represents the frequency of the concentration-dependent consumption reaction (1/s). Considering the complexity of the model, the oxygen concentration at the outer boundary of the culture medium is set to 1 mol/m^3^, the diffusion coefficient in the culture medium is 2 × e^−9^ m^2^/s, the diffusion coefficient for EC@GelMA encapsulation is 2.4 × e^−10^ m^2^/s, the diffusion coefficient for BMSE-SEAs construct is approximately 1 × e^−8^ m^2^/s, and the reaction frequency on the construct surface is set to 1.6 s^−1^.

The mathematical model serves as an abstract representation of the *in vitro* environment and therefore involves certain simplifying assumptions. For instance, cell density is assumed to be uniform across the spheroid, implying that local oxygen consumption depends solely on position and oxygen concentration. Consequently, *R* remains constant regardless of spatial position.

### 5.13 *In vitro* swelling ratio and in *vivo* degradation test

Cylindrical GelMA hydrogels (6 mm diameter, 2 mm height) were fabricated. The initial wet weight of each hydrogel was recorded as (W_0_), and the wet weight after incubation for predefined time points (0, 3, 6, 12, 24, and 48 h) was recorded as (W_t_). The swelling ratio was calculated as (W_t_/W_0_ × 100%). For *in vivo* degradation, cylindrical hydrogels were implanted subcutaneously into the dorsal region of nude mice. At predetermined time points (1, 4, 7, and 14 days) post-implantation, the mice were euthanized and the transplanted hydrogels, along with the surrounding tissues, were harvested. After carefully removing the outer fibrous capsule and surface liquid, the wet weight of each retrieved hydrogel was recorded. The degradation rate was expressed as the ratio of wet weight to the initial wet weight at each time point.

### 5.14 Subcutaneous implantation

BMSC-SEAs were constructed following the above-described method and cultured in chondrogenic medium for 3 weeks to form BEC. Then, GelMA hydrogel or EC@GelMA mixture was coated (4 layers) on the surface of some of the BEC samples to form BEC-GelMA, and BEC-EC@GelMA, respectively. Immediately after encapsulation, constructs from the three groups (BEC as control, BEC-GelMA, and BEC-EC@GelMA) were implanted subcutaneously into the dorsal region of nude mice (n = 5 per group). Samples were harvested at 4- and 8-weeks post-implantation to evaluate the stability of cartilage regeneration. All specimens were carefully stripped of the surrounding connective tissue, and overall images were collected at different time points.

### 5.15 Microcomputed tomography (Micro‐CT) analysis

Samples harvested at 4- and 8-weeks post-implantation were subjected to micro-CT scans (μCT-80, Switzerland) according to the manufacturer's default setup. The machine was operated in the high-resolution scanning mode to quantify the extent of ossification. 2D projections were automatically reconstructed using the inbuilt Micro-CT software, while the bones were visualized by observing the 3D isosurfaces. Quantitative morphometric analyses were performed to determine the BV, BV/TV, BMC, and BMD by Analyze 12.0 software (PerkinElmer, USA).

### 5.16 Histological and immunohistochemical staining

After micro-CT analysis, all engineered tissues harvested above, were subjected to histological and immunohistochemical examinations as described previously [7]. samples were fixed in 4% paraformaldehyde, decalcified using 10% ethylenediamine tetraacetic acid, embedded in paraffin, and sectioned. The regenerated cartilage samples were sectioned into 5 μm slices for staining. H&E, SO&FG, and Masson's trichrome staining were performed. And COL II (SC-52658, 1:100, Santa Cruz, USA) and OCN (23418-1-AP, 1:50, Proteintech, China) in the engineered cartilage were detected via the IHC to observe the cartilage-specific structure and ECM deposition of the regenerative cartilage and osteoblasts, respectively. The sections were counterstained with hematoxylin to produce a brown precipitate at the antigenic site.

We performed double immunofluorescence staining for HIF-1α, VEGF, CD31, α-SMA and PGP9.5 in different groups’ sections. After deparaffinization in xylene and rehydration through a graded ethanol series (100% to 75%), antigen retrieval was performed by heating the sections in EDTA buffer (pH 9.0) using a microwave oven. The sections were then blocked with 10% goat serum for 30 min at room temperature. Then, anti-CD31 (ABB00001, 1: 100, Huilan Biotech, China), anti-α-SMA antibody (ET1607-53, 1: 50, HuaBio, China), and anti- PGP9.5 (14730-1-ap, 1: 200, Proteintech) were used as primary antibodies for overnight incubation at 4 °C. Fluorescein isothiocyanate-conjugated with the corresponding secondary antibodies (1: 200) for 1 h at room temperature in the dark, and the nuclei were counterstained with DAPI. The stained slides were scanned by using 3D Histech Pannoramic MIDI (3D HISTECH Ltd., Hungary). In addition, the CD31/α-SMA and PGP9.5 staining images were analyzed using Fiji (ImageJ2) to quantitatively analyze the number of blood vessels/field.

### 5.17 Statistics analysis

Data were analyzed with SPSS version 25.0 (IBM Inc., USA), R version 4.30 (https://www.R-project.org), and GraphPad Prism version 8.0 (GraphPad Software, Inc., USA). All values are reported as the means ± standard deviations. Statistical significance was defined as a two-sided p-value or adjusted *P* ≤ 0.05 (n.s., *P* > 0.05; *, *P* ≤ 0.05; **, *P* < 0.01; ***, *P* < 0.001). For all quantitative experiments, the exact sample size (n) and the number of independent biological replicates are explicitly stated in the figure legends or corresponding Methods subsections. Comparisons between two groups were performed using Student's t-test. For multiple-group comparisons, one-way analysis of variance (ANOVA) followed by Bonferroni's post hoc test was used. All results were confirmed in at least three independent experiments.

## CRediT authorship contribution statement

**Bao-xing Tian:** Writing – original draft, Methodology, Investigation, Formal analysis, Conceptualization. **Hui-lin Sun:** Methodology, Investigation, Formal analysis. **Bao-shuai Bai:** Visualization, Validation, Formal analysis, Data curation. **Chen Jin:** Visualization, Methodology, Investigation. **Qian-yi Wang:** Methodology, Formal analysis. **Guang-dong Zhou:** Writing – review & editing, Project administration, Investigation. **Xiao-xi Lin:** Visualization, Supervision. **Bin Wang:** Validation, Supervision. **Dong Lei:** Writing – review & editing, Funding acquisition, Data curation, Conceptualization. **Meng-jie Hou:** Writing – review & editing, Visualization, Investigation, Funding acquisition, Formal analysis, Conceptualization.

## Data availability

The data that support the findings of this study are available from the corresponding author upon reasonable request.

## Ethics approval and consent to participate

In this study, all animals received humane care, in accordance with the National Institutes of Health Guide for the Care and Use of Laboratory Animals (NIH Publications No. 8023, revised 1978). All protocols for experiments in animals were approved by the Animal Care and Experimental Committee of Shanghai Rat&Mouse Biotech Co.,Ltd (220250914 (13)). All samples were radiation sterilized for subsequent tests.

## Declaration of competing interest

The authors declare that the research was conducted in the absence of any commercial or financial relationships that could be construed as a potential conflict of interest.

## Figures and Tables

**Table:** 

Abbreviation	Full name
α-SMA	α-smooth muscle actin
BEC	BMSC-engineered cartilage
BMC	Bone mineral content
BMD	Bone mineral density
BMP-2	Bone morphogenetic protein-2
BMSCs	Bone marrow mesenchymal stem cells
BV	Bone volume
BV/TV	Bone volume fraction
COL II	Type II collagen
DAB	3,3-diaminobenzidine tetrahydrochloride
DEGs	Differential expression genes
DiD	1,1′-Dioctadecyl-3,3,3′,3′-Tetramethylindodicarbocyanine, 4-Chlorobenzenesulfonate Salt
DiO	3,3′-dioctadecyloxacarbocyanine perchlorates
DMEM	Dulbecco's Modified Eagle Medium
EC	Ear chondrocyte
ECM	Extracellular matrix
EGDE	Ethylene glycol diglycidyl ether
FBS	Fetal bovine serum
FTIR	Fourier transform infrared
FPKM	Fragments per kilobase of transcript per million mapped reads
GelMA	Gelatin methacryloyl
GSEA	Gene Set Enrichment Analysis
GO	Gene Ontology
H&E	Hematoxylin and eosin
IGF-I	Insulin-like growth factor-I
IHC	Histological and immunohistochemical
KEGG	Kyoto Encyclopedia of Genes and Genomes
OCN	Osteocalcin
PBS	Phosphate-buffered saline
PCA	Principal component analysis
PGP9.5	Protein gene product 9.5
PPI	Protein-protein interaction
SEM	Scanning electron microscopy
SF	Silk fibroin
SF-SEAs	SF super elastic absorbent sponges
SO&FG	Safranin O and fast green
TEMED	N,N,N′,N′-tetramethylethylenediamine
TGF-β1	Transforming growth factor beta-1
XRD	X-ray diffraction
