# Supplementary material for: Constructing hypoxia and bioshield inhibits neurovascularized endochondral ossification for stable cartilage regeneration
Source: Bioact Mater. 2026 Oct 10;68:988–1003. doi: 10.1016/j.bioactmat.2026.08.030 (PMC13677771; doi:10.1016/j.bioactmat.2026.08.030)
Supplement: Supplementary file 1 [file mmc1.docx]

**Supplementary Figure**

**Figure S1. Hydrophilicity and Compressive properties of dry SF-SEAs scaffolds.** A) A) Mass of SF-SEAs scaffolds in dry and hydrated states. B) Representative compressive stress-strain curves. C) Cyclic compression test results. Data are mean ± SD. ***, *P* < 0.001.

**Figure S2. Preparation of BMSC-SEAs construct.** A) Gross view of an acellular SF-SEAs scaffold. B) Gross view of BMSC-SEAs constructs.

**Figure S3. Characterization of photo-crosslinkable GelMA hydrogel.** A) 5% (w/v) GelMA solution. B) UV light exposure (405 nm) for photopolymerization. C) Photopolymerized GelMA hydrogel. D) Swelling ratio of GelMA hydrogel *in vitro*. E) Gross view of residual GelMA hydrogel after subcutaneous implantation in nude mice. F) *In vivo* degradation ratio of GelMA hydrogel after subcutaneous implantation in nude mice. G) *In vivo* stability of GelMA hydrogel coatings on SF-SEA scaffolds in rabbit model.

**Figure S4. Quantitative comparison of chondrocyte numbers.** A) Live/dead staining of ECs in EC@GelMA. B) Additional live/dead staining confirming EC viability. C) Comparison of chondrocyte numbers between encapsulation and traditional construction methods. *, *P* < 0.05.

**Figure S5. Quality control of transcriptomic sequencing data.** A) FPKM values before and after normalization. B) Box plot of normalized gene expression across samples.

**Figure S6. Expression patterns of common DEGs.** A) Heatmap showing expression levels of the 1,017 intersected DEGs across the three experimental groups.

**Figure S7. GSEA of key signaling pathways and immunofluorescence for HIF-α and VEGF.** A, B) Enrichment of the MAPK signaling pathway in the BMSC-SEAs-EC@GelMA group compared to the BMSC-SEAs-GelMA group and the BMSC-SEAs group, respectively. C, D) Enrichment of the TGF-β signaling pathway in the BMSC-SEAs-EC@GelMA group compared to the BMSC-SEAs-GelMA group and the BMSC-SEAs group, respectively. E, F) Enrichment of the BMP receptor binding signaling pathway in the BMSC-SEAs-EC@GelMA group compared to the BMSC-SEAs-GelMA group and the BMSC-SEAs group, respectively. G, H) Enrichment of the ECM‑receptor interaction pathway in the BMSC-SEAs-EC@GelMA group compared to the BMSC-SEAs-GelMA group and the BMSC-SEAs group, respectively. I, J) immunofluorescence for HIF-α. K, L) Immunofluorescence for VEGF.

**Figure S8. Biosafety assessment of in vivo implantation.** A) H&E staining of major organs (heart, liver, spleen, lung, and kidney) eight weeks post-implantation. Scale bar: 200 μm.

**Figure S9. Immunofluorescence analysis of vascular and neural invasion inhibition.** A, B) Double immunofluorescence staining for CD31 (green) and α-SMA (red). Nuclei are counterstained with DAPI (blue) C, D) Immunofluorescence staining for PGP9.5 (green). Nuclei are counterstained with DAPI (blue). Red scale bar: 1 mm, white scale bar: 100μm.
